# Non-Invasive Detection of SARS-CoV-2 Antigen in Saliva versus Nasopharyngeal Swabs Using Nanobodies Conjugated Gold Nanoparticles

**DOI:** 10.3390/tropicalmed7060102

**Published:** 2022-06-13

**Authors:** Manal Kamel, Sara Maher, Hanan El-Baz, Faten Salah, Omar Sayyouh, Zeinab Demerdash

**Affiliations:** 1 Immunology Department, Theodor Bilharz Research Institute, Giza 12411, Egypt; baher_ronny@hotmail.com (M.K.); helbaz77@gmail.com (H.E.-B.); fatensdm215@gmail.com (F.S.); zeinabdem@hotmail.com (Z.D.); 2 Infection Control Department, Theodor Bilharz Research Institute, Giza 12411, Egypt; o.sayyouh@tbri.gov.eg

**Keywords:** SARS-CoV-2, diagnosis, ELISA, nanobodies, saliva

## Abstract

The development of sensitive, non-invasive tests for the detection of severe acute respiratory syndrome coronavirus-2 (SARS-CoV-2) antigens is imperative, and it is still challenging to manage the extent of infection throughout the population. Here, we designed and optimized a sandwich enzyme-linked immunosorbent assay (ELISA) protocol for SARS-CoV-2 S1 antigen detection in saliva. Both saliva samples and nasopharyngeal swabs were collected from 220 real-time quantitative polymerase chain reaction (RT-qPCR)-confirmed positive and negative cases. S1 protein receptor-binding domain (RBD) nanobodies were efficiently conjugated with 40 nm gold nanoparticles (AuNPs) and employed as antigen detection probes in the developed system, while recombinant S1 monoclonal antibodies (S1mAbs) were employed as antigen capture probes. After checkerboard assays and system optimization, the clinical samples were tested. In saliva, the developed ELISA system showed the highest sensitivity (93.3) for samples with cycle threshold (Ct) values ≤ 30; interestingly, high sensitivity (87.5 and 86%) was also achieved for samples with Ct values ≤ 35 and ≤40, respectively, compared with 90, 80 and 88% sensitivity rates for nasopharyngeal swabs with the same categorized Ct values. However, the specificity was 100%, and no cross-reactions were detected with Middle East respiratory syndrome coronavirus (MERS-CoV) or SARS-CoV antigens. These results reveal that our protocol could be established as an efficient and sensitive, non-invasive diagnostic tool for the early detection of SARS-CoV-2 infection using easily collectable saliva samples.

## 1. Introduction

Coronavirus disease 2019 (COVID-19) is a major public health problem caused by severe acute respiratory syndrome coronavirus 2 (SARS-CoV-2) [1]. In March 2020, it was declared a pandemic by the World Health Organization (WHO) [2]. Patients with COVID-19 have varied clinical features that range from mild symptoms to severe life-threatening manifestations, and the growing trend of infected cases is not yet under control [3]. Despite this, a gradual ease of COVID-19 restrictions is now seen in many countries. The success of this “post-pandemic exit strategy” needs continued surveillance of the virus, as well as the prevalence evaluation among population and serology tests, which are valuable tools for these purposes [4]. RT-qPCR is the current gold standard for detection. However, it is restricted by its high cost, its time-consuming nature, the requirement of high technical expertise and non-specific false-positive and false-negative amplifications, in addition to the uncomfortable nasopharyngeal swab process [5]. Now, during this post-pandemic period, mass screening is essential, and screening tests should be affordable, sensitive and non-invasive. Antigen detection is an actual detector for the current infection with high sensitivity and specificity [6]. Monoclonal antibodies (mAbs) are successfully applied in the development of different antigen-based ELISA systems showing excellent results, but their large size and complex structure may represent various defects, such as limited tolerability [7]. These obstacles could be overcome by using single-domain antibodies known as nanobodies (Nbs). Nbs are the variable domains of camelid heavy-chain antibodies without light chains, small fragments (15 kDa) with effective antigen-binding units, and exhibit a less complex structure. So, they have been successfully produced in bacterial cells at much lower cost and with no production limits [8]. With their revolutionized structural biology, Nbs have arisen as outstanding features for diagnostic and therapeutic applications [9]. Recently, multiple research studies have reported Nbs’ neutralizing effect against SARS-CoV-2 spike receptor binding domain (RBD) as well as their potential application in ELISAs for the efficient detection of SARS-CoV-2 S1 antigen [10,11,12].

Currently, naso-/oropharyngeal swabs are the main recommended upper respiratory tract specimen types for COVID-19 diagnostic testing, although the use of saliva for the diagnosis of the disease has been recently suggested [13]. Using naso- and oropharyngeal swabs has several limitations, such as discomfort for the patient or bleeding in patients with thrombocytopenia or any other coagulation disorders. These drawbacks can limit the use of swabs, especially in serial monitoring or mass test programs [14]. The use of saliva or sputum represents an easy, fast and cheap way to collect samples, allowing widespread testing to be conducted.

Nanomaterials improve the sensitivity and the detection limit of traditional ELISAs as they provide additional binding sites for detection antibodies and improve the signal intensity [15]. Gold nanoparticles, due to their high chemical stability and large surface-area-to-volume ratio, could be used to design highly sensitive diagnostic probes [16]. Nanobodies conjugated with gold nanoparticles (AuNPs-Nbs) could be achieved by physical adsorption or covalent conjugation which represent more stable conjugate that with much more stability when using covalent conjugation and could be applied successfully in several applications [17].

In this study, we developed a sandwich-based ELISA by the employment of S1 recombinant Nbs conjugated with gold nanoparticles as detector probes and S1 mAbs as capture probes for the sensitive and accurate detection of SARS-CoV-2 spike protein (S1) in both saliva samples and nasopharyngeal swabs. Our results were compared to those of RT-qPCR for the same patients. This protocol could offer an alternative non-invasive, sensitive and specific antigen detection assay to detect COVID-19 early infection in the population, which is vitally important for disease-spreading prevention and control.

## 2. Methods

### 2.1. Clinical Samples

Sample collection was carried out at the COVID-19 outpatient clinic at TBRI (July–November 2021; variant of concern Delta B.1.617.2), in accordance with relevant guidelines and precautions of WHO concerns and regulations. Nasopharyngeal samples from symptomatic patients (males and females with average ages of 35–65 years old) were collected by trained nurses using the universal viral transport (UVT) system. A flexible mini-tip swab was passed through the patient’s nostril till the posterior nasopharynx, left for several seconds and then slowly removed while rotating. The swab was placed in sterile viral transport medium (3 mL total volume), sealed securely and sent for RT-qPCR testing after preserving an aliquot at −80 °C for testing using the developed ELISA. Un-stimulated saliva samples were self-collected by the patient early in the morning. Patients were asked to spit repeatedly into the sterile cups, which were securely closed and preserved rapidly at −80 °C till use.

### 2.2. Detection of SARS-CoV-2 Using RT-qPCR

RT-qPCR was performed for all collected nasopharyngeal swabs by the national reference lab using One-step Real Time RT-PCR Master Mixes kits (Thermo Fisher, Waltham, MA, USA) for SARS-CoV-2 typical N and ORF1ab target genes. The assay was performed according to the manufacturer’s protocol of the kit.

### 2.3. Conjugation of SARS-CoV-2 S1-RBD Nanobodies (RBD-Nbs) with Colloidal Gold NANOPARTICLES (AuNPs)

The covalent conjugation of Nbs with colloidal gold nanoparticles was carried out using a gold conjugation kit (ab154873; Abcam, Cambridge, UK) according to the manufacturer’s protocol. The gold nanoparticles (40 nm) included in this kit had a protective surface coat table to withstand the most extreme conditions, which allowed an ultra-stable conjugation product to be obtained. Gold-conjugated Nbs were then kept at 4 °C till use after being checked by a UV-Vis-spectrophotometer (Jenway 6305; Cambridge, UK).

### 2.4. Testing the Reactivity of AuNPs-Nbs Probes and Free Nbs against SARS-CoV-2 S1 Antigen

The relative reactivity of the conjugate probes (AuNPs-Nbs) and free nanobodies were tested by an indirect ELISA. A 96-well microtiter plate was coated with 100 μL serial dilutions of recombinant human coronavirus SARS-CoV-2 spike glycoprotein S1 (ab 288546 (B.1.617.2); Abcam, Cambridge, UK), starting with 2-fold serial dilutions from 200 ng to 1.5 ng per well in carbonate buffer (0.1 M, pH 9.8), and incubated overnight at 4 °C. The plate was washed 3 times with washing buffer (0.01 M PBS, pH 7.4) and then blocked with 200 μL/well of 0.1% BSA (Sigma Aldrich, MO, USA) in 0.1 M PBS, pH 7.4, for 2 h at 37 °C. Volumes of 100 μL of three different concentrations (5, 2.5 and 1.25 μg/mL) of AuNPs-Nbs and free Nbs were added. Following incubation for 1 h at 37 °C, the plate was washed three times as described above. A volume of 100 μL of goat anti-llama horseradish peroxidase (HRP) conjugate (ab112786; Abcam, Cambridge, UK) (1/10,000 in PBS, pH 7.4) was added to each well. The plate was then incubated for 1 h at RT. After washing, 100 μL of stabilized chromogen, TMB solution (3,3′,5,5′-tetramethylbenzidine; Elabscience), was added for 20 min, and the reaction was stopped by the addition of 50 µL of 1 M HCl. The optical density (OD) value was measured at 450 nm using an ELISA microplate reader (Multiscan FC; Thermo Fisher) without reference wavelengths.

### 2.5. Development and Optimization of Sandwich ELISA

A checkerboard analysis for testing the best concentrations of both capture and detector probes was employed by using three serial concentrations of recombinant S1mAbs, the capture proteins (10, 5 and 2.5 μg/mL), in coordination with three serial concentrations of AuNPs-Nbs, the detector proteins (5, 2.5 and 1.25 μg/mL). The sandwich ELISA was standardized by using five confirmed RT-qPCR-positive and five confirmed RT-qPCR-negative cases. Each step was tested in triplicate under the same conditions. Polystyrene microtiter plates (flat-bottomed 96-well plates; M 129 a; Dynatech) were coated with 100 µL/well of SARS-CoV-2 S1mAb (No. ab281311; Abcam, Cambridge, UK) at 3 different concentrations (10, 5 and 2.5 μg/mL) in 0.1 M carbonate buffer, pH 9.6, and incubated overnight at 4 °C. The wells were washed 3 times with 0.01 M PBS, pH 7.2. The remaining active sites in the wells were blocked with a blocking buffer at 200 µL/well, 5% skim milk (*w*/*v* DW). Plates were incubated overnight at 4 °C and then washed 3 times with 200 µL/well of 0.01 M PBS/Tween-20, pH 7.2. A volume of 100 µL/well of saliva (used as such without dilution) or nasopharyngeal swabs solutions was added and incubated for 90 min at 37 °C. The wells were washed 3 times with washing buffer; then, 100 µL/well of AuNPs-Nbs (three concentrations were tested: 5, 2.5, and 1.25 μg/mL) was added, which was followed by incubation for 2 h at room temperature (RT). The wells were then washed 3 times, and 100 µL of goat anti-llama HRP conjugate (ab112786; Abcam, Cambridge, UK) was added at two different dilutions (1:1000 and 1:10,000), according to the manufacturer. Plates were incubated for 2 h at RT and then washed 5 times with the same washing buffer described above. A volume of 100 µL/well of TMB substrate solution was added, and the plates were incubated for 15–30 min at RT in the dark; then, 50 µL/ well of stopping solution (2 M H_2_SO_4_) was added. The absorbance was checked at 450 nm against a blank using an ELISA reader (Multiscan FC; Thermo Fisher). The assessment of the cross-reactivity of the developed SARS-CoV-2 S1 sandwich ELISA was examined by using both MERS-CoV and SARS antigens. 

### 2.6. Evaluation Using Clinical Samples

The stored saliva and nasopharyngeal swabs, both positive (n = 120) and negative (n = 100), were examined in triplicate using the optimized conditions of the developed ELISA system to evaluate its sensitivity and specificity.

### 2.7. Statistics

Statistical analyses were executed using SPSS software version 22. Independent *t*-tests were utilized to study the statistical differences in the absorbance between AuNPs-Nbs and Nbs-only groups. A linear regression analysis and Pearson’s correlation coefficient were used to fit the relationship and correlate the changes in absorbance with the changes in Ag concentration. Receiver operating characteristic (ROC) analyses were executed to determine the best cutoff value that discriminated positively from negative accounting for the overall sensitivity and specificity levels of the technique. Graph Pad Prism version 8 was used to obtain the scattered plots of positive, negative and blank values. Qualitative data were described as number and percentage. Kappa as a measure of agreement was calculated. A *p*-value less than or equal to 0.05 was considered statistically significant. All tests were two-tailed.

## 3. Results

### 3.1. Clinical Samples Characterization

A total of 220 specimens were primarily examined with RT-qPCR. Positive (n = 120), with different ranges of Ct values (≤30, ≤35 and ≤40), and negative (n = 100) samples were determined. All saliva and nasopharyngeal swabs were stored at −80 °C and were tested using the developed ELISA system. 

### 3.2. Characterization and Testing of Reactivity of AuNPs-Nbs as Well as Free Nbs against SARS-CoV-2 S1 Recombinant Antigen

**Figure 1:**
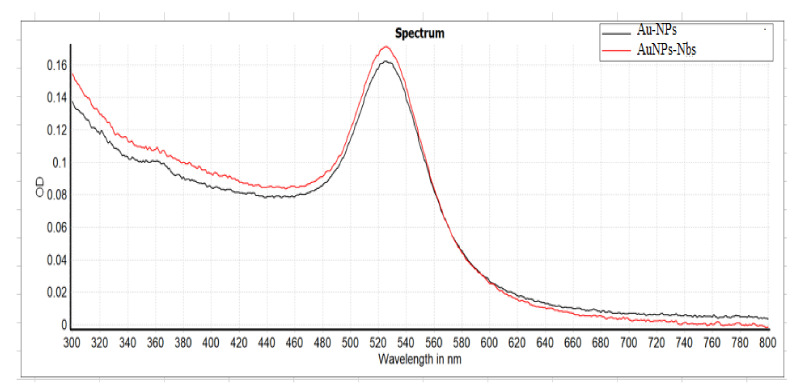
UV-Vis spectra of colloidal gold nanoparticles and gold-conjugated nanobodies. Black: colloidal gold solution. Red: gold-conjugated nanobodies.

**Figure 2:**
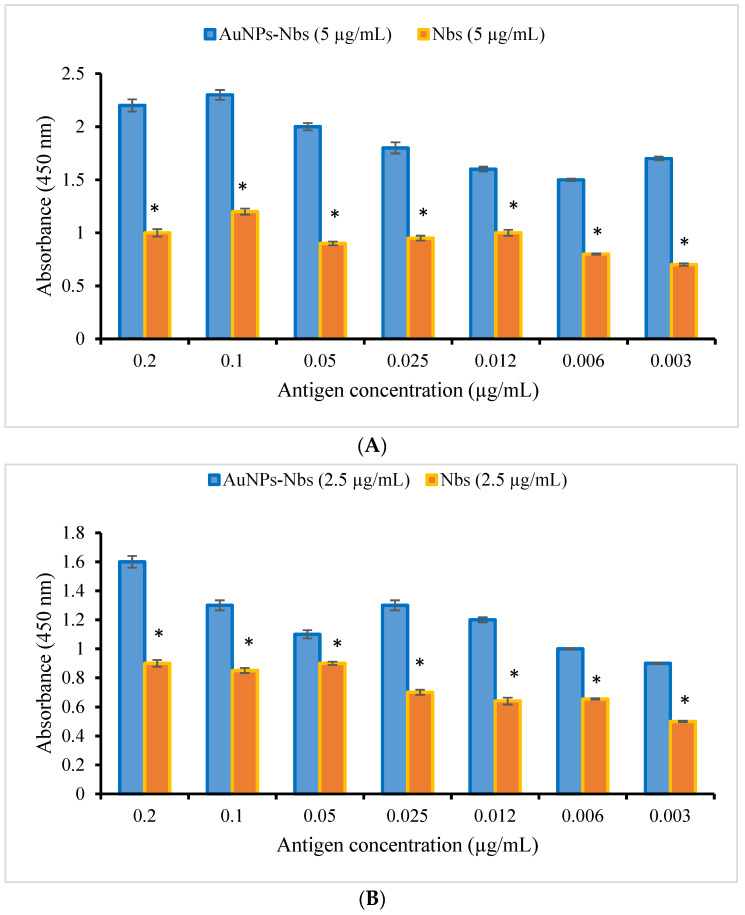

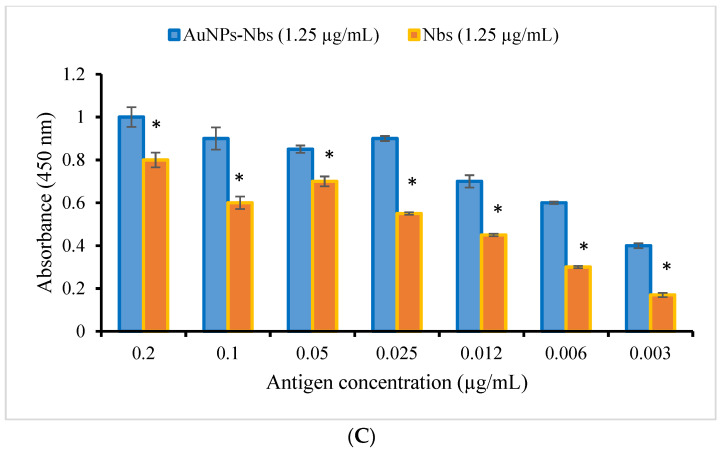
Test results of the reactivity of three different concentrations ((**A**) 5 µg/mL, (**B**) 2.5 µg/mL and (**C**) 1.25 µg/mL) of both SARS-CoV-2 S1 (RBD) nanobodies conjugated with gold nanoparticles (AuNPs-Nbs) and free nanobodies against different concentrations of recombinant SARS-CoV-2 S1 antigen. * represents significant difference (*p* < 0.000) as compared with the corresponding AuNPs-Nbs at each antigen concentration. Each bar represents mean ± standard error of the mean.

**Figure 3:**
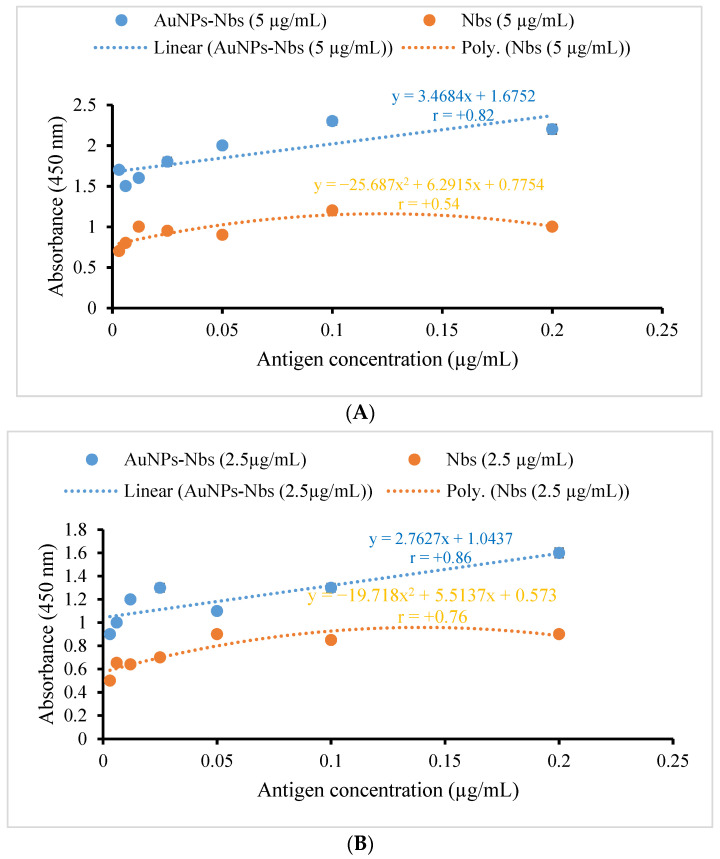

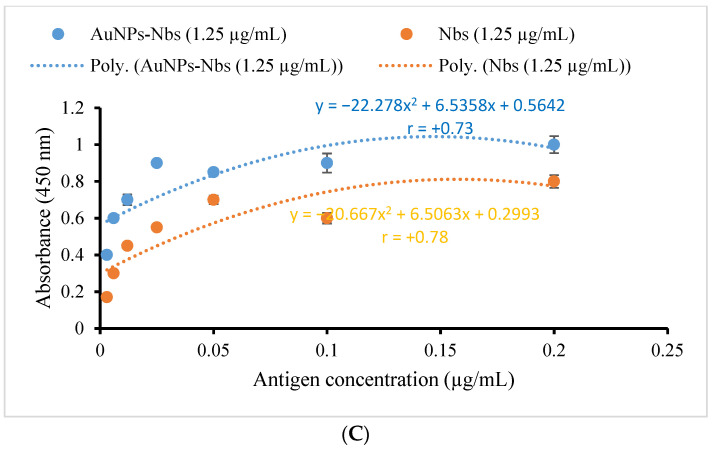
Fitting relationship of the absorbance with antigen concentrations when using three different concentrations of AuNPs-Nbs ((**A**) 5 µg/mL, (**B**) 2.5 µg/mL and (**C**) 1.25 µg/mL) as well as free Nbs. r: Pearson’s correlation coefficient between absorbance and Ag concentration.

Following the conjugation procedures, the AuNPs-Nbs conjugates were checked using a UV-Vis spectrophotometer; the peaks were shifted after conjugation from 522 nm to 528 nm (Figure 1). Furthermore, the reactivity of conjugated AuNPs-Nbs and free nanobodies were assessed with an indirect ELISA. The absorbance at different concentrations of S1 antigen using different concentrations of AuNPs-Nbs (1.25, 2.5 and 5.0 µg/mL) as well as Nbs alone are displayed in Figure 2. The figure shows that at each antigen concentration, there was a significant increase in the absorbance reading by using AuNPs-Nbs, as compared with using Nbs alone. Direct relationships were recorded between the absorbance reading and the antigen concentration in the presence of Nbs alone or conjugated with AuNPs. The highest correlation coefficient (r = 0.86) was reported in the case with the 2.5 µg concentration of AuNPs–Nbs (Figure 3).

### 3.3. Optimization of Sandwich ELISA

**Figure 4:**
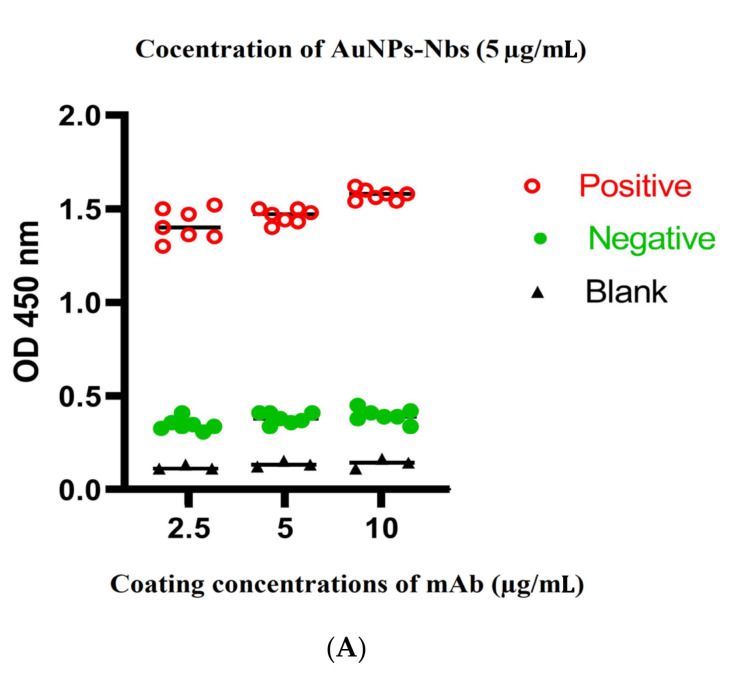

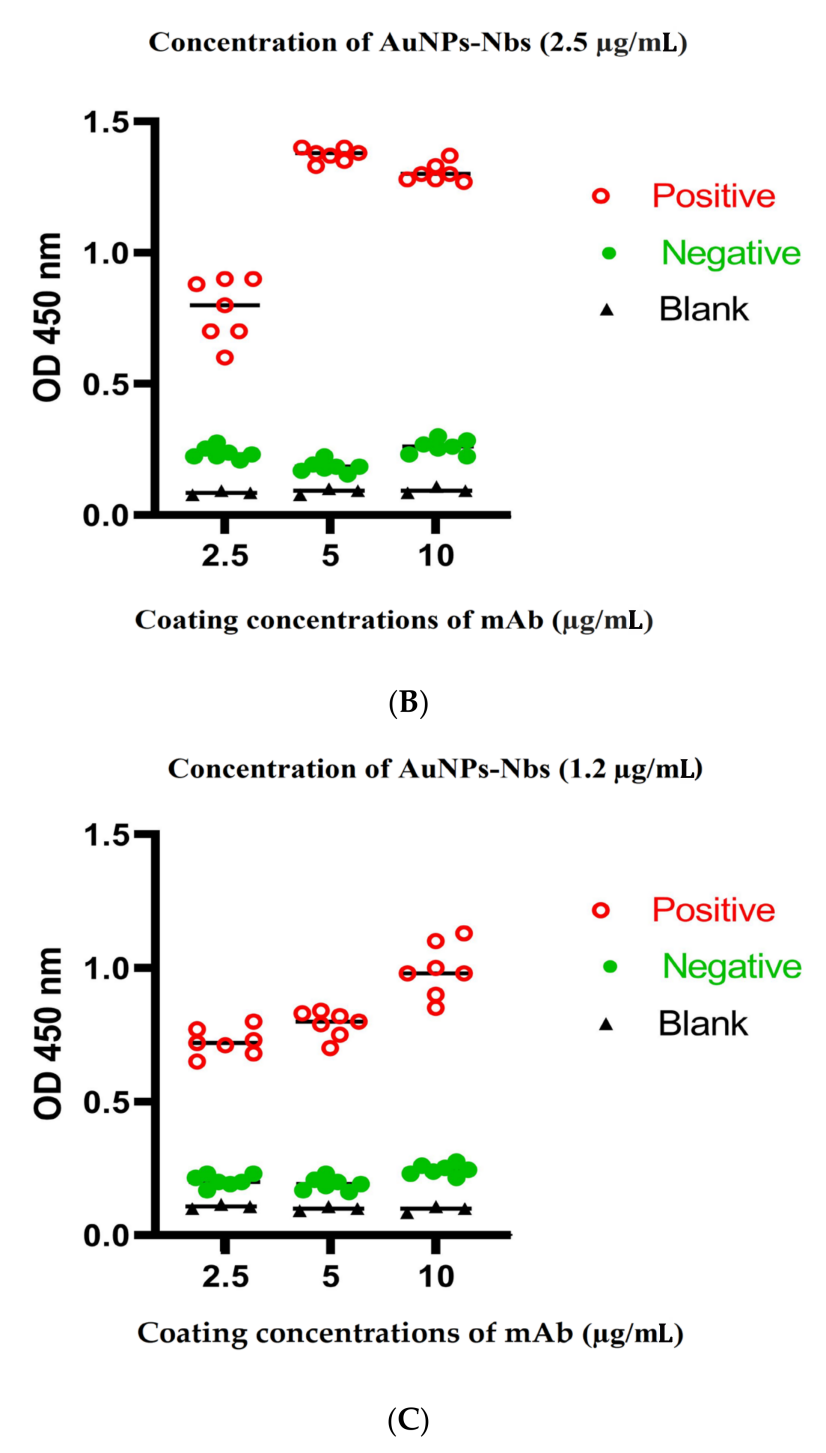
Optimization of sandwich ELISA system using three different concentrations of SARS-CoV-2 S1 mAbs (capture antibodies) (10, 5 and 2.5 µg/mL) and three concentrations of gold-conjugated nanobodies (AuNPs-Nbs) as detector probes: (**A**) AuNPs-Nbs concentration of 5 µg/mL, (**B**) AuNPs-Nbs concentration of 2.5 µg/mL and (**C**) AuNPs-Nbs concentration of 1.2 µg/mL. **NB**: The optimum pairing concentrations were obtained by using S1mAb at 5 µg/mL concentration and AuNPs-Nbs at 2.5 µg/mL concentration.

Checkerboard analyses were performed using three different concentrations of coating probes (S1 mAbs), 10, 5 and 2.5 μg/mL. On the other hand, detecting probes (AuNPs-Nbs) were applied at 5, 2.5 and 1.25 μg/mL. All tests were performed under the same conditions and incubation periods. Two dilutions of anti-llama HRP conjugate were tested (1:1000 and 1:10,000). RT-qPCR-confirmed positive and negative saliva samples and nasopharyngeal swabs were applied to obtain optimum conditions for the subsequent assessment of the tested samples. The optimum sandwich system should ensure the highest OD ratio between positive and negative readings, as well as the lowest OD ratio between negative and blank readings. These criteria were achieved using 5 μg/mL S1 mAbs (capture antibodies) and 2.5 μg/mL AuNPs-Nbs (detector antibodies) at 1:10,000 dilution for anti-llama HRP conjugate (Figure 4).

### 3.4. Cutoff Value and Evaluation of the Developed Sandwich ELISA System

**Figure 5:**
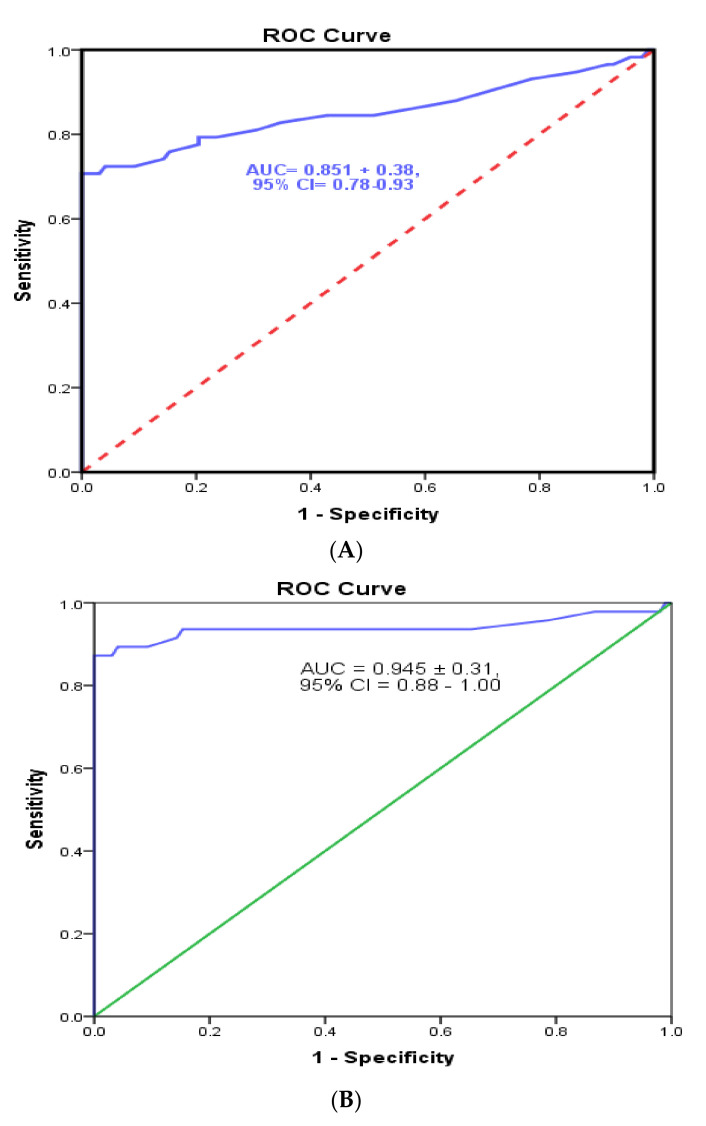
Receiver operating characteristic (ROC) analyses of the developed sandwich ELISA for the detection of SARS-CoV-2 S1 antigen in nasopharyngeal swabs (**A**) as well as in saliva samples (**B**).

**Figure 6:**
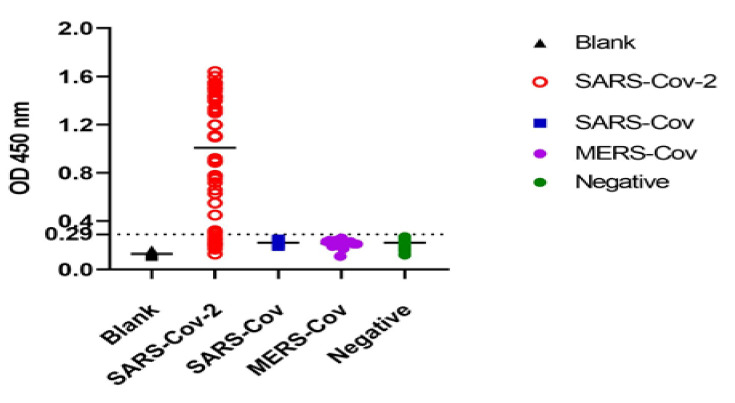
Assessment of reactivity of the developed SARS-CoV-2 S1 sandwich-based ELISA on RT-PCR-positive and -negative saliva samples using S1 mAbs as capture antibodies and nanobodies-conjugated gold nanoparticles (AuNPs-Nbs) as a detector antibodies. MERC-Cov and SARS-CoV antigens were used to test cross reactivity.

This study was conducted on 120 RT-qPCR-confirmed SARS-CoV-2 positive cases with different Ct values and 100 RT-qPCR-confirmed negative cases. Saliva samples and nasopharyngeal swabs of the same cases were collected and tested using our optimized sandwich ELISA. Cross reactions with SARS-CoV and MERS-CoV antigens were also tested. ROC analyses were executed to estimate the best cutoff values that offered maximum sensitivity and specificity. For nasopharyngeal swabs, the area under the ROC curve equaled 0.85 ± 0.38, with a 95% confidence interval (CI) of 0.78–0.94; the cutoff value that gave 87.5% overall sensitivity and 0% 1-specificity (i.e., 100% specificity) was 0.27 (Figure 5A). For saliva samples, the present technique revealed an area under the ROC curve equal to 0.945 ± 0.31 and a 95% confidence interval (CI) of 0.88–1.00 and showed that the cutoff value that gave 88.7% overall sensitivity and 0% 1-specificity (i.e., 100% specificity) was 0.29 (Figure 5B). No cross-reactions were detected with SARS-CoV nor MERS-CoV antigens (Figure 6). Furthermore, we observed and analyzed data on the performance of our ELISA system with various RT-qPCR Ct values of positive-tested samples (i.e., ≤30, ≤35 and ≤40). Categorizing the samples according to the Ct values, a higher sensitivity (93.3%) was achieved in saliva for positive samples with Ct values ≤ 30, and a high sensitivity also obtained in samples with Ct ≤ 35 and ≤40 (Table 1). Furthermore, sensitivity in accordance with the Ct values of nasopharyngeal positive samples showed high values but lower than those for saliva samples (90%) with Ct ≤ 30 but slightly higher than those with Ct ≤ 40 (88%) (Table 2). Cohen’s Kappa test was used to evaluate the test agreement and inter-rater reliability between the results of the developed ELISA and RT-qPCR tests in both saliva and nasopharyngeal swabs according to the Ct value, indicating a near-perfect agreement in saliva samples as well as nasopharyngeal swabs (Table 2 and Table 3).

## 4. Discussion

Controlling the spread of COVID-19 relies on diagnostic tests, particularly for identifying asymptomatic infection. RT-PCR is considered the standard for SARS-CoV-2 detection. However, there are many limitations to using PCR in diagnosis, including low sensitivity and the complex processes that could not meet the urgency of the early diagnosis of the vast numbers of suspected cases, as well as patients’ discomfort during nasopharyngeal swabs [18]. Many commercial kits are available for antibodies detection but with uncertain performance [19]. However, earlier diagnosis by antigen-based detection system in the acute phase of the disease is much better for accurate results. Recently, a few antigen kits have been established to detect SARS-CoV-2 with various efficiencies [20,21,22]. However, to our knowledge, no ELISA techniques have been published for the antigen assessment of SARS-CoV-2 except for the work by Adnan et al. (2021), which presented 92.9 % sensitivity with Ct values ≤ 30 but lower sensitivity with Ct values ≤ 35, 40 [23].

Here, we developed and optimized an advanced sandwich ELISA protocol for the accurate and sensitive detection of SARS-CoV-2 S1 antigen in saliva based on the employment of gold-conjugated nanobodies (detector antibodies) and S1 mAbs (capture antibodies). Following the functionalization of gold nanoparticles with nanobodies, we evaluated the reactivity of these conjugates by comparing them with free nanobodies against serial concentrations of SARS-CoV-2 S1 recombinant antigen, and we obtained a significant increase in the absorbance reading by using AuNPs-Nbs, as compared with using Nbs alone. These results reinforced our hypothesis and gave us the green light for proceeding in the designing of this system. Furthermore, a checkerboard analysis was performed by testing different concentrations of S1mAbs and AuNPs-Nbs to optimize our system; then, we tested both saliva samples and nasopharyngeal swabs under the same conditions. We evaluated the sensitivity and specificity of our protocol by testing saliva samples and nasopharyngeal swabs from 120 confirmed RT-qPCR-positive and 100 RT-PCR-confirmed negative cases. By using the developed and optimized ELISA system for testing saliva samples, we obtained a higher sensitivity (93.3%) when the Ct values of the positive samples were ≤30 and, interestingly, a high sensitivity also for Ct values of ≤35, 40 (87.5% and 86%). On the other hand, the nasopharyngeal swabs showed slightly lower sensitivity than saliva, except for Ct values of ≤40, with a sensitivity of 88%. Our results show an impressive elevation in sensitivity when testing samples with different viral loads up to Ct ≤ 40; to our knowledge, this is the first ELISA protocol to achieve such results. These elevated sensitivity results could be strongly correlated with the employment of nanobodies and their conjugation to gold nanoparticles. Nanobodies, with their small sizes and stability, exhibit an advantage over antibodies as sensitive probes in ELISAs for COVID-19 diagnosis, as represented in the recent work by Girt et al. (2021) [12]. These results are in agreement with our previous work (Kamel et al. (2016)) [24], as well as other research works, where the conjugation of colloidal gold nanoparticles to antibodies allowed a highly sensitive and specific sandwich ELISA system to be obtained compared with the conventional ELISA [25,26]. Interestingly, the overall sensitivity and specificity of the test were nearly similar in both saliva and nasopharyngeal swabs samples, ensuring that the use of the more convenient saliva samples could be a validated replacement of nasopharyngeal swabs. Moreover, by obtaining near-perfect agreement between our developed ELISA system and RT-qPCR, we could employ this system to reduce the burden of molecular tests, especially for regular testing of health coworkers as well as the hospital of Theodor Bilharz Research Institute (TBRI). We conclude that our newly developed protocol could be established as a new, reliable, sensitive and non-invasive screening test for the diagnosis of SARS-CoV-2 infection using easily collectable saliva samples. 

### Limitation of the Study

We did not test this developed system on growing variants of concern (VoCs) such as Omicron. Further research for the development and optimization of nanobodies-based ELISA systems for the diagnosis of the S1 antigen of predominant VoCs is recommended.

## Figures and Tables

**Table 1:** Variation in sensitivity and specificity of the developed ELISA system in both saliva and nasopharyngeal specimens depending on Ct value.

	Sensitivity	Specificity	PPV	NPV	Total Accuracy
Ct ≤ 30					
In saliva	93.30%	100%	100%	98%	98.4
In nasopharyngeal swabs	90%	100%	100%	97%	97.6
Ct > 30- ≤ 35					
In saliva	87.50%	100%	100%	95%	96.2
In nasopharyngeal swabs	80%	100%	100%	92%	93.9
Ct > 35- ≤ 40					
In saliva	86%	100%	100%	93%	95.1
In nasopharyngeal swabs	88%	100%	100%	94%	95.8

**Table 2:** Inter-rater reliability between developed ELISA system results (in saliva samples) and RT-qPCR according to Ct value.

Ct Value			RT-qPCR		Kappa Coefficient
			P	N	
Ct ≤ 30	ELISA	P	28	0	0.955 *
			21.9%	0.0%	
		N	2	98	
			1.6%	76.6%	
Ct > 30- ≤ 35	ELISA	P	35	0	0.908 *
			25.9%	0.0%	
		N	5	95	
			3.7%	70.4%	
Ct > 35- ≤40	ELISA	P	43	0	0.889 *
			30.1%	0.0%	
		N	7	93	
			4.9%	65.0%	

Cohen’s Kappa test was used to evaluate the test agreement. * *p* value < 0.001. Data are represented as numbers and percentages of total.

**Table 3:** Inter-rater reliability between developed ELISA system results (in nasopharyngeal swabs) and RT-qPCR according to Ct value.

Ct Value			RT-qPCR		Kappa
			P	N	
Ct ≤ 30	ELISA	P	27	0	0.932 *
			21.3%	0.0%	
		N	3	97	
			2.4%	76.4%	
Ct > 30- ≤ 35	ELISA	P	32	0	0.848 *
			24.2%	0.0%	
		N	8	92	
			6.1%	69.7%	
Ct > 35- ≤ 40	ELISA	P	44	0	0.905 *
			30.6%	0.0%	
		N	6	94	
			4.2%	65.3%	

Cohen’s Kappa test was used to evaluate the test agreement. * *p* value < 0.001. Data are represented as numbers and percentages of total.

## Data Availability

All data are presented in the manuscript only.
